# Editor's Letter-Box

**Published:** 1899-10-21

**Authors:** 


### Editor's Letter-Box.

[Our Correspondents are reminded that prolixity is a great bar to publication, and that brevity of style and conciseness of statement greatly facilitate early insertion.]

Mr. F. Eardley Wilmot, R.N., secretary of the Church of England Temperance Society, in the course of a long letter commenting upon an article which appeared in The Hospital, October 7th, entitled "Teetotalism and Philanthropy," writes as follows: Taking our own society, we have three permanent homes for female inebriates and two shelter-homes for short periods, besides nine labour yards, to four or five of which there are regular homes attached for dealing with male cases. Turning to other societies, there is the home run by the British Women at Duxhurst, near Reigate, and by the Women's Total Abstinence Union at Sydenham, which are the direct outcome of the practical work of temperance people. I might go on indefinitely adding, almost, to the catalogue of practical work which is taken up by the temperance party, and which clearly shows that it is neither "a party with one idea, or that it is devoid of charity."

*** We are very glad to publish these statements, although they do not seem to us to touch the fact on which our comments were founded. What we said was this: "We find from the annual report of the inspector of the retreats licensed under the Inebriates Acts that there are but fourteen such establishments, and that there is an utter absence of any retreat for the accommodation of male patients who are either destitute or are possessed of only strictly limited means." And we do not gather that our correspondent denies that this is true.—Ed. *T. H.*

